# The Radiologic Evaluation and Clinical Significance of Glenohumeral Bone Loss in Anterior Shoulder Instability

**DOI:** 10.3390/jcm13247708

**Published:** 2024-12-17

**Authors:** Matthew A. Zinner, Eric V. Neufeld, Andrew D. Goodwillie

**Affiliations:** 1 Northwell Health, New Hyde Park, NY 11040, USAeneufeld1@northwell.edu (E.V.N.); 2 Department of Orthopaedic Surgery, Long Island Jewish Medical Center/North Shore University Hospital, New Hyde Park, NY 11030, USA

**Keywords:** glenohumeral bone loss, shoulder instability, glenoid track

## Abstract

Glenoid and humeral bone loss is associated with a high incidence of recurrent shoulder instability and failure of arthroscopic stabilization procedures. However, the radiographic evaluation of bony Bankart and Hill–Sachs injuries continues to pose a diagnostic challenge, and a universally accepted optimal method of measurement is lacking. The purpose of this review is to summarize the advantages and disadvantages of various techniques and imaging modalities available for measuring glenoid bone loss in shoulder instability, including conventional roentgenography, 2-dimensional and 3-dimensional computed tomography (CT), and magnetic resonance imaging (MRI). We also review the concepts of engaging “on-track” and “off-track” Hill–Sachs lesions. Finally, we highlight the clinical importance of obtaining accurate determinations of bone loss by the various methods available, as it can affect surgical decision making and the appropriate procedure required to ensure shoulder stability is adequately restored.

## 1. Introduction

The shoulder is the most often dislocated joint in the body, with greater than 90% of shoulder instability occurring anteriorly. Dislocation most often results from a traumatic injury such as a fall or collision [1]. The rate of anterior dislocation is 23.1 per 100,000 person years and is three times more common in males than females. The overall distribution of anterior shoulder instability favors younger patients with a median age of 35 and the highest incidence in those under 20 years old [2]. The high rate of instability of the shoulder joint is multifactorial and can be partially attributed to its substantial range of motion. The muscles of the rotator cuff comprise the primary dynamic stabilizers of the glenohumeral joint, while the glenoid labrum, negative intra-articular pressure, and glenohumeral ligaments serve as static stabilizers, particularly at the extremes of motion [3]. It is this delicate interplay between the bony architecture and surrounding soft tissues that provides the shoulder with the greatest range of motion of any joint; however, imbalance can ultimately lead to instability.

Identifying osseous defects in patients with traumatic anterior shoulder instability is paramount, as both humeral and glenoid bone loss can increase the rate of recurrent instability and failed operative management if not addressed [4,5,6]. A universal gold-standard method to calculate bone loss is currently lacking, although there are many techniques available to surgeons to assess and measure glenohumeral bone loss. An accurate measurement of bone loss is of prime importance, as it can affect surgical decision making to ensure long term success in the surgical management of these complex injuries.

## 2. Glenoid Track Theory

Bankart lesions, defined as anteroinferior labral injuries with or without osseous avulsion, are considered the most common defect following anterior shoulder dislocations, with an intraoperative prevalence up to 97% percent [7]. Surgical treatment of instability typically includes arthroscopic repair of the Bankart lesion. However, the presence of concurrent bone loss at the time of dislocation both from the glenoid and the humeral head—in the form of a Hill–Sachs lesion—can lead to surgical failure [8].

Glenoid bone loss occurs in the form of a bony Bankart lesion: a fracture of the anteroinferior aspect of the glenoid rim alongside the capsulolabral injury [9]. Nakagawa et al. found that just over one-third of patients presented with a glenoid defect at the time of primary instability [10]. First-time anterior dislocations on average resulted in 6.8% of glenoid bone loss [11]. Bone loss can also occur with a compression fracture of the posterosuperolateral humeral head that results from impaction during anterior shoulder dislocation. This is referred to as a Hill–Sachs lesion and is identified in 47% and 90% of patients of first-time dislocators [7,12].

It is important to distinguish between engaging and non-engaging Hill–Sachs deformities. An engaging Hill–Sachs lesion is when the humeral head defect engages the glenoid rim. This occurs when the defect is parallel to the anterior glenoid rim, causing it to engage when the shoulder is abducted and externally rotated. Contrarily, a non-engaging Hill–Sachs lesion features a non-parallel humeral head defect that does not engage the glenoid rim in functional positions. Whether or not a lesion is engaging can be predicted by its size, location, and the degree of glenoid bone loss [13].

**Figure 1:**
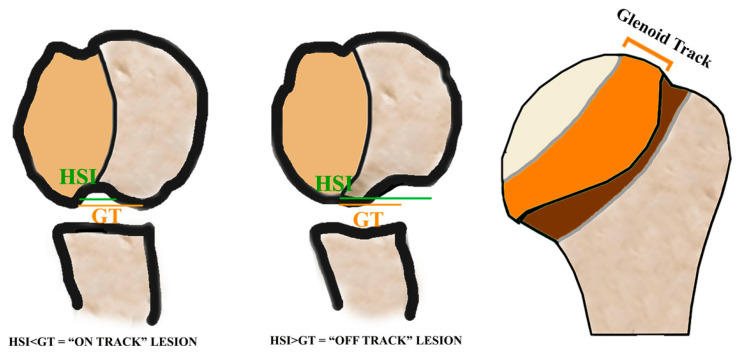
Glenoid track. HSI: Hill–Sachs index; GT: glenoid track [16].

The glenoid track concept can be helpful in evaluating patients with associated bone loss and can ultimately help guide surgical management (Figure 1). The contact point between the glenoid and humeral head is dynamic throughout the range of motion. Yamamoto et al. found that as the arm is raised, the point of contact moves from the inferomedial to the superolateral portion of the posterior articular surface of the humeral head [14]. The glenoid track is the various points of contact between the humeral head and glenoid throughout the anatomic arc of motion. The glenoid track (GT) can be determined by the formula (GT = 0.83 *D* − *d*), where “D” is the diameter of the glenoid (D) and “*d*” is the amount of anterior glenoid bone loss compared to the contralateral arm [15]. A factor of 83% is utilized due to the insertion of the rotator cuff on a portion of the glenoid track.

**Figure 2:**
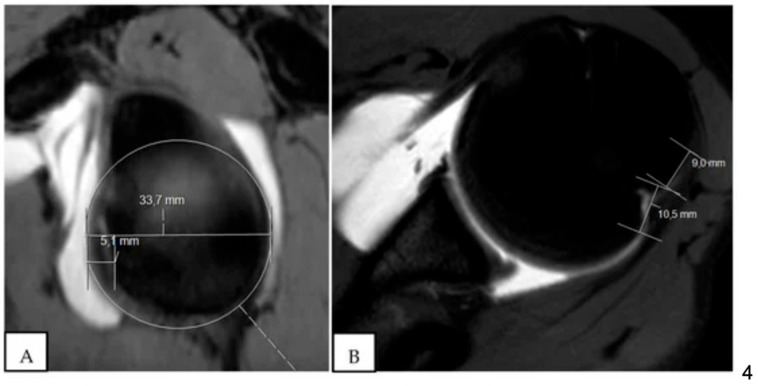
On-track lesion on magnetic resonance imaging (MRI) arthrography. (**A**) In this case, the glenoid track is equal to 23 mm (33 − 5 × 0.83 = 23). (**B**) The Hill–Sachs index (HSI) is 19.5 mm (10.5 + 9). It is an on-track lesion because the glenoid track (23 mm) is greater than the HSI (19.5 mm) [16].

**Figure 3:**
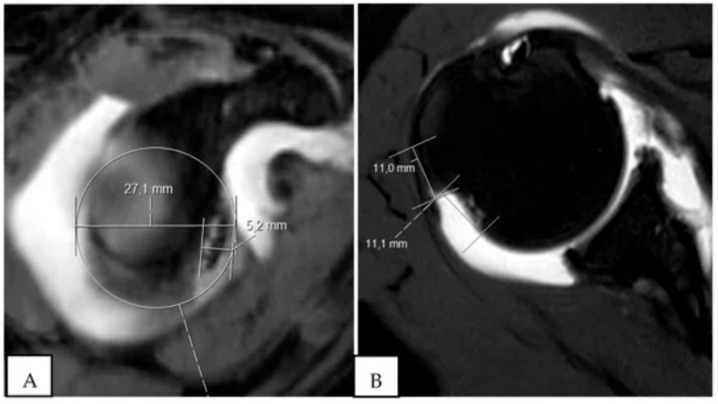
Off-track lesion on magnetic resonance imaging (MRI) arthrography. (**A**) In this case, the glenoid track is equal to 18.17 mm (27.1 – 5.2 × 0.83 = 18.17). (**B**) The Hill–Sachs index (HSI) is 22.1 mm (11.1 + 11). It is an off-track lesion because the glenoid track (18.17 mm) is less than the HSI (22.1 mm) [16].

The concept of the glenoid track is a unifying theory that takes into account the size and degree of glenoid bone loss but crucially also factors in the specific location of the defect to determine if the lesion will engage. A Hill–Sachs lesion that is larger than the glenoid track is considered an “off-track” deformity (Figure 2 and Figure 3). Yamamoto et al. proposed that these “off-track” Hill–Sachs lesions are located outside the contact area of the glenoid and humeral head, placing them at higher risk of engagement and thus recurrent instability [14]. As glenoid bone loss increases, the contact area decreases, thus increasing the likelihood that a Hill–Sachs lesion is “off-track”. This distinction is critical to operative decision making, as patients with engaging lesions are considered to be at higher risk of recurrent instability and thus warrant surgical consideration [13,17]. In fact, Kurokawa et al. recommended that the glenoid track should be utilized to assess engaging Hill–Sachs lesions rather than dynamic intraoperative assessment [18]. They believed that assessing the lesions intraoperatively may lead to overdiagnosing engaging lesions due to ligamentous instability, allowing the humeral head to shift anteriorly. Thus, they proposed that the “true” definitions of engaging Hill–Sachs were those with lesions that exceeded the size of the glenoid track or that engaged during intraoperative assessment following Bankart repair. In a follow-up study of 100 shoulders with recurrent anterior instability, 94 had Hill–Sachs lesions and 7 were found to be engaging based on the glenoid track definition [18].

It is essential to understand that osseous abnormalities involving the glenoid or humerus do not occur in isolation but often interact as a bipolar phenomenon. In patients with Hill–Sachs lesions, there is a synergistic effect leading to a direct relationship between the degree of glenoid bone loss and instability even in patients with intermediate-sized Hill–Sachs lesions [19,20]. Provencher et al. determined that the size and morphology of Hill–Sachs lesions is directly related to the amount of glenoid bone loss. Patients who presented with less glenoid bone loss had narrower and deeper Hill–Sachs lesions with less humeral head surface area loss, while their counterparts with increased glenoid bone loss had wider and shallow Hill–Sachs lesions comprising a significantly larger percentage of the humeral head [21].

## 3. Imaging

### 3.1. Roentgenography

The radiologic evaluation of shoulder instability should begin with plain radiographs. The standard initial set includes an anteroposterior (AP) view in the plane of the scapula (i.e., a “true” AP or Grashey view), a scapular “Y” lateral view, and an axillary view. Orthogonal imaging is crucial in assessing whether the humeral head is concentrically reduced and can be helpful in determining direction of instability.

**Figure 4:**
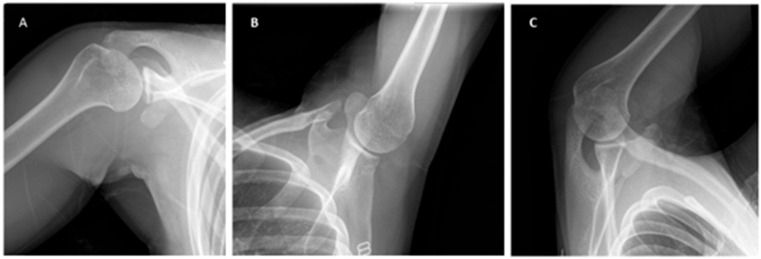
(**A**) Stryker notch, (**B**) West Point, and (**C**) Bernageau views.

Additional imaging can include the Stryker notch, West Point, and Bernageau views (Figure 4). The Stryker notch view is obtained with the patient supine and the cassette under the affected shoulder. The patient’s ipsilateral hand is placed on top of the head with their fingers towards the occiput. The X-ray is shot with the beam tilted ten degrees cephalad and centered over the coracoid process [22]. The Stryker notch view can help identify Hill–Sachs lesions with a reported sensitivity of 81% and specificity of 100% [23,24]. The West Point view allows for a tangential view of the anteroinferior glenoid rim. The view is obtained with the patient prone and the affected shoulder raised with a bump approximately 8 cm above the table with the forearm dangling free. The cassette is held against the superior aspect of the shoulder, and the beam is aimed at the axilla at a 25-degree angle downward and medially [22]. The Bernageau view is obtained with the patient standing with the arm at maximal forward flexion, usually 160 degrees. The cassette is placed in contact with the thorax at an angle of 70 degrees, and the X-ray is shot 30 degrees caudally and centered on the scapular spine [25]. Edwards et al. found that 79% of osseous abnormalities of the glenoid may be identified utilizing the Bernageau view [26]. Murachovsky et al. described a method of using the Bernageau view to calculate glenoid bone loss. The anterior-to-posterior distance of the glenoid was measured and then compared to the contralateral side to determine the percentage of bone loss. This method was reproducible with an interobserver correlation coefficient of 0.81 and demonstrated comparable results to 3D CT [25].

The advantage of radiographs when evaluating for glenohumeral bone loss is their accessibility and affordability. However, in many cases, plain radiographs alone are insufficient to assess the presence and degree of bone loss. Yiannakopoulos et al. found that up to 60% of bony lesions are missed when diagnosis relies solely upon plain radiographs [27]. Moreover, some of the aforementioned views can be technically challenging to the unfamiliar radiographer and are not routinely obtained at the time of an initial evaluation of shoulder instability. Considering that many orthopedic surgeons obtain a computed tomography (CT) scan regardless of the radiographic findings, it can be argued that the practicality of the aforementioned special views may be limited.

### 3.2. Computed Tomography (CT)

**Figure 5:**
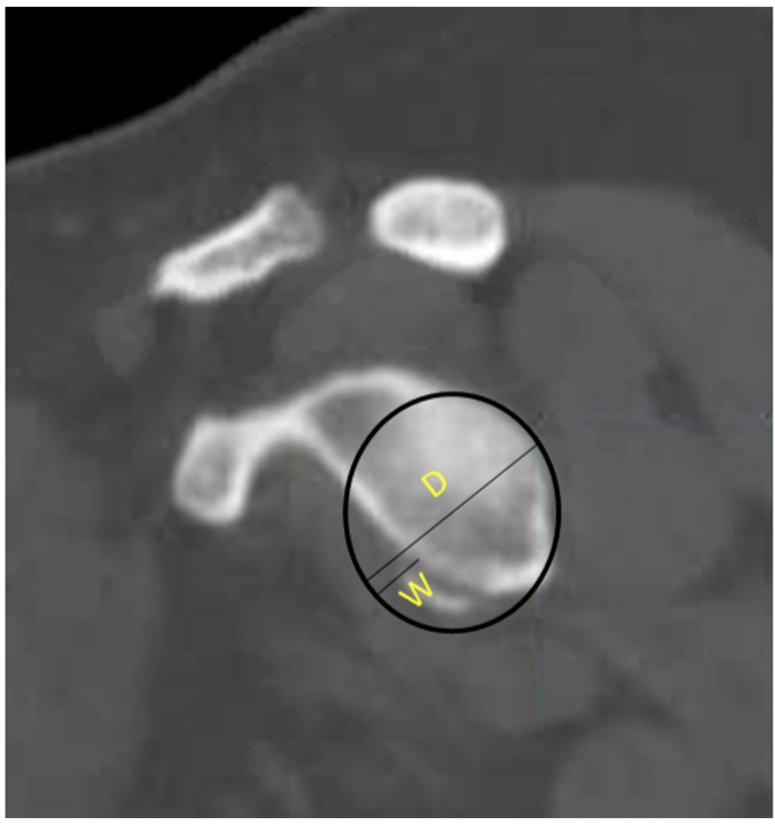
Best-fit-circle method of measuring glenoid bone loss. D = Diameter of best-fit circle, W = Width of defect, W/D = % of bone loss.

**Figure 6:**
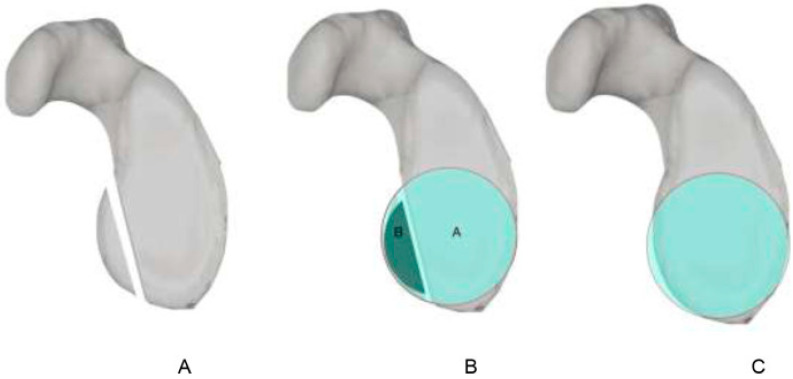
Pico method [35]. (**A**) CT slice of glenoid with anterior bone loss, (**B**) Best fit circle with area of anterior bone loss, (**C**) Best fit circle from contralateral glenoid. A: Best fit circle from contralateral glenoid, B: Area of anterior glenoid bone loss.

CT is currently considered the gold standard for detecting and quantifying glenohumeral bone loss [24,28,29]. Griffith et al. found that CT had a sensitivity of 78% and specificity of 93% for identifying the presence of glenoid bone loss [30]. CT is also particularly useful for quantifying the degree of glenoid bone loss, which is of paramount clinical significance given its positive relationship with the failure of isolated Bankart repair. Two common methods of assessing glenoid bone loss are the best-fit circle technique (Figure 5) or the measurement of glenoid width utilizing the Griffith index and the Pico method (Figure 6) [31]. Both methods involve digitally subtracting the humeral head to provide an en face view of the glenoid.

The Griffith method involves simultaneous CT imaging of both shoulders. The maximum width of the glenoid is calculated by measuring a line perpendicular to the vertical axis of the glenoid. The width of the injured side is compared to the contralateral side and expressed as a percentage loss [32].

The Pico method of quantifying glenoid bone loss relies on the fact that the inferior aspect of the glenoid approximates a circle [33,34]. The area of the circle can be determined based on the contralateral side or utilizing the intact posteroinferior margins of the injured glenoid. The amount of glenoid bone loss is then expressed as the surface area of the circle compared to the contralateral side [28].

While both methods are effective, the literature has demonstrated that comparison to the contralateral side is more accurate [36]. Zappie et al. found that utilizing the CT Pico method had superior inter-user reliability compared to MRI. The surface area calculation method also demonstrated an excellent interobserver reliability of 0.95 [31]. In addition to calculating glenoid bone loss, CT has demonstrated an excellent ability to identify on-track and off-track Hill–Sachs lesions, with Burns et al. demonstrating a sensitivity of 92% and specificity of 100% for predicting engagement with the shoulder in abduction and external rotation [37].

It is important to note that in a recent international consensus statement, 95% of experts agreed that the 3-dimensional (3D) CT is the most accurate method to identify and quantify glenoid bone loss. And while they felt that any of the described methods are adequate, an en face view of the glenoid using 3D CT is the single most accurate imaging method currently described [38].

### 3.3. Magnetic Resonance Imaging (MRI)

**Figure 7:**
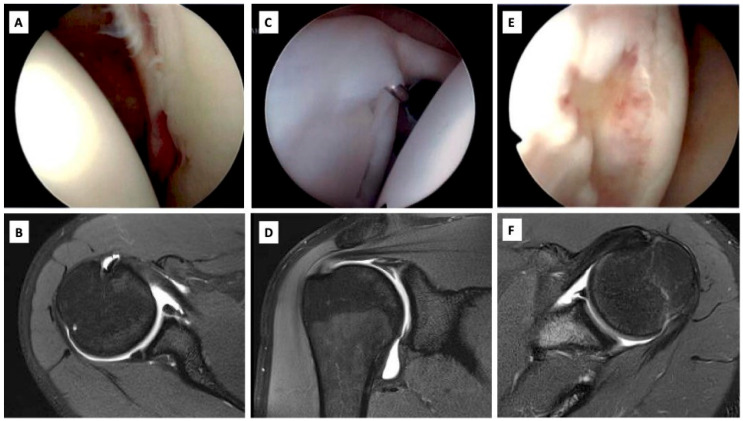
(**A**) Intraoperative image of Bankart lesion. (**B**) MR imaging of Bankart lesion. (**C**) Intraoperative image of SLAP tear. (**D**) MR imaging of SLAP tear. (**E**) Intraoperative image of Hill Sachs. (**F**) MR imaging of Hill–Sachs.

MRI serves a valuable role in the management of anterior shoulder instability, allowing for assessment of the soft-tissue structures of the shoulder. Depending on the etiology of the injury, glenohumeral instability may be associated with Bankart lesions, humeral avulsion of the glenohumeral ligament (HAGL), glenoid labral articular disruption (GLAD) lesions, anterior labroligamentous periosteal sleeve avulsion (ALPSA), and injury to the rotator cuff [17]. All of these injuries can be assessed on MRI and are important to identify, as they may significantly alter the operative plan (Figure 7). Because MRI is often already a part of the diagnostic algorithm for shoulder instability, there is an increased interest in its utility in identifying and quantifying glenohumeral osseous abnormalities.

**Figure 8:**
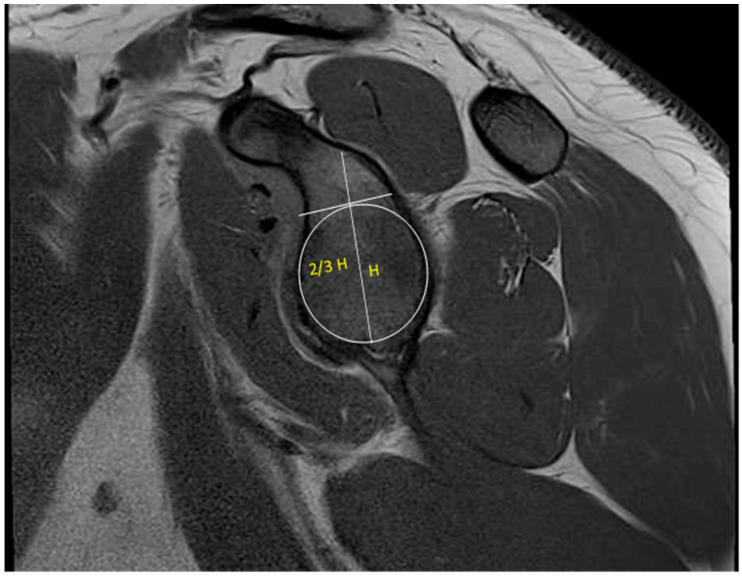
Two-thirds height perfect-circle method.

There is ample evidence that MRI is capable of quantifying the degree of glenoid bone loss [39]. Gyftopoulos et al. demonstrated that MRI could be accurately used to determine glenoid bone loss, with results comparable to CT and 3D CT [40]. Makovicka et al. recently proposed a new MRI-based technique for calculating glenoid bone loss. Instead of utilizing the borders of the glenoid to create a perfect circle, they suggested using two-thirds of the glenoid height to establish the diameter of a perfect circle of the glenoid (Figure 8). They found that this method produced similar results for the area of the perfect circle with superior reproducibility and consistency, allowing for a more objective assessment of glenoid bone loss [41]. MRI has also been able to identify on-track versus off-track Hill–Sachs deformities with a sensitivity of 72% and specificity of 88% [40]. Gyftopoulos et al. described a method of assessing glenoid bone loss utilizing the aforementioned circle method on 3D MRI. They found a direct relationship between the degree of glenoid bone loss and engaging Hill–Sachs lesions [42]. Stillwater et al. also demonstrated that 3D MRI achieved similar results in measuring humeral head loss, Hill–Sachs size, percent of humeral head loss, and percent of glenoid bone loss [43]. While these studies contained a limited number of participants, they suggest it may be possible to utilize 3D MRI in place of CT or 3D CT. This would help reduce the radiation exposure to patients and lower costs by requiring one fewer advanced imaging modality.

### 3.4. Ultrasound

While less common, there is a renewed interest in utilizing ultrasound for the assessment of osseous glenohumeral defects. Ultrasound does have multiple inherent benefits including its low cost, accessibility, rapidity, and ability to conduct a dynamic exam. Simão et al. described three different techniques: an anterior transverse approach with the patient supine and the arm adducted, an axillary approach with the patient supine and the arm in abduction and external rotation, and a transverse posterior approach with the patient seated and the arm adducted. The arm was internally and externally rotated to allow for a dynamic exam [44]. In a systematic review, Vopat et al. found that ultrasound had a sensitivity of 74–96% and a specificity of 60–95% for identifying Hill–Sachs lesions [24]. Because ultrasound relies on the experience and technique of the sonographer, it has been shown to have a relatively low interobserver agreement (0.19–0.40) [44].

## 4. Clinical Significance of Glenohumeral Bone Loss

Glenoid bone loss occurs in up to 90% of patients with recurrent glenohumeral instability. Given its role in determining optimal treatment, obtaining an accurate measurement is essential. Sugaya et al. initially proposed subdividing glenoid bone loss into three categories: large, defined as greater than 20% of the glenoid surface area; medium, between 5 and 20%; and small, less than 5% [45]. Extensive research has sought to determine the critical value of bone loss that predicts failure of arthroscopic Bankart repair alone and recurrence of instability. In a cadaveric study, Yamamoto et al. found that glenoid loss exceeding 20% of the length or 26% of its width was enough to induce instability [46]. These findings have classically led to many surgeons employing bony augmentation of the glenoid in patients with 20–25% glenoid loss [17,28]. However, more recent studies have demonstrated that patients may experience clinically significant deficits and an increased risk of failed soft-tissue repair alone with as little as 13.5% of glenoid bone loss [47,48].

Hill–Sachs lesions and humeral head bone loss are also important to consider, as they may be present in up to 93% of patients with recurrent glenohumeral instability [27]. Similar to glenoid bone loss, there is a direct relationship between the failure of arthroscopic Bankart repairs and recurrent instability with the increasing size of Hill–Sachs lesions [49]. Lesions that involve less than 20% of the humeral head are rarely considered clinically significant. Contrarily, defects greater than 40% of the humeral head are almost universally clinically significant and contribute to recurrent instability [19,50]. The intermediate-sized group of lesions between 20% and 40% poses a management dilemma. To better assess these intermediate defects, one must understand the synergistic relationship between humeral and glenoid bone loss based on both their size and location. Provencher et al. suggested a set of absolute and relative indications for the operative management of Hill–Sachs lesions. Absolute indications were (1) lesions > 30–40% of the humeral head with chronic dislocation or recurrent anterior instability or (2) reverse lesions with >20–40% of humeral head articular surface involvement and symptoms of posterior instability, catching, or pain. Relative indications were (1) lesion > 20–35% of the humeral head with glenoid engagement on examination, (2) lesion > 20% of the articular surface and signs of humeral head engagement on examination, (3) lesion > 10–25% of the humeral head that does not remain well centered in the glenoid fossa after arthroscopic stabilization, or (4) reverse lesion with humeral head cartilage involving 10–30% of the humeral head with symptoms of posterior instability, catching, or pain [19].

In many cases, the management of an engaging Hill–Sachs lesion is isolated glenoid bone augmentation in the form of a Latarjet procedure, iliac crest autograft, distal clavicle autograft, or distal tibia allograft. All these procedures increase the size of the glenoid track, helping to prevent engagement of the Hill–Sachs lesion [51]. In the rare cases of isolated humeral bone loss without associated glenoid bone loss, surgeons may address the Hill–Sachs lesion directly via osteochondral allograft transfers or more commonly the utilization of a remplissage technique to fill the defect [52,53]. The indications for osteochondral allograft transfers for isolated humeral lesions are not clearly defined, but successful outcomes have been achieved in patients with Hill–Sachs lesions, reverse Hill–Sachs lesions, and post-arthroscopy chondrolysis [54]. The remplissage technique involves a posterior capsulodesis and infraspinatus tenodesis and then using sutures to pull the released capsule and infraspinatus tendon into the Hill–Sachs defect. This procedure can be performed in isolation or conjunction with an arthroscopic Bankart repair [53].

## 5. Current Limitations

Despite the current understanding of glenohumeral bone loss and the ability to ensure precise measurements of bone loss, clinical implementation can be difficult secondary to logistical barriers. Instability patients typically present with imaging already obtained, which notoriously is limited to post-reduction orthogonal views that do not allow for adequate evaluation of glenoid and humeral bone loss. Moreover, most routine CTs are not performed with humeral head subtraction, especially those patients that obtain this imaging study during their initial evaluation in the emergency department. Repeat imaging is left to the orthopedic surgeon’s discretion but exposes the patient to greater radiation.

Logistically, even with adequate CT imaging, understanding on which specific cut to perform measurements and where to draw perfect circles to determine bone loss may not be obvious. There is clinical judgement as to how to determine the exact CT cut that is most representative of the glenoid’s surface area. In addition, minor technique variations between the radiologist and orthopedic surgeon can lead to a few millimeter differences in bone loss measurement. This may lead to false over- or under-estimates of critical bone loss and hence may ultimately affect surgical planning. While hardly insurmountable, these realities pose a hurdle to the routine utilization of these methods in clinical practice.

Considering the logistical challenges of many of these approaches, Lederman et al. recently proposed a simplified ratio for estimating glenoid bone loss. Their calculations demonstrated that the only measurements needed are the glenoid height and length of the glenoid defect. They found that a ratio of defect length to glenoid height of 0.5 corresponds to 12% bone loss, and a ratio of 0.56 corresponds to 19.1% bone loss [55]. This technique may simplify the ability to determine surgical treatments and measurement of subcritical bone loss. However, it does not address the criticism raised by Moroder that these techniques fail to consider the 3D nature of glenoid bone loss and the differences in patients‘ native glenoid anatomy [56].

Other limitations also exist with the assessment and management of Hill–Sachs deformities. In the 2021 consensus statement, 95% of experts felt that current imaging systems poorly quantified and classified Hill–Sachs deformities, and there was no consensus on the best management and rehabilitation of these injuries [38]. This remains an area with significant room for additional investigation.

The continued integration of artificial intelligence (AI) into imaging platforms has the potential to help address these current shortcomings. A well-trained AI system may have the ability to select for the ideal CT or MRI cut to calculate glenoid bone loss. Additionally, the ability of an AI system to perform the calculations would standardize measurement techniques, thereby reducing inter-user variability. Future research should compare the ability of AI systems to identify glenohumeral bone loss on advanced imaging compared to human observers and correlate those interpretations with intraoperative findings.

## 6. Summary

The topic of glenohumeral bone loss in anterior shoulder instability is an area of ongoing research. Significant strides have been made in recent years in understanding the role of glenoid bone loss and engaging Hill–Sachs lesions in persistent instability and failed soft-tissue Bankart repair. The workup of these patients typically begins with a thorough history and physical exam focusing on the number of instability events, the timing since dislocation, and the presence of engagement on exam. Most clinicians will obtain a series of plain films that may include the Stryker notch, West Point, or Bernageau views to better assess for specific osseous abnormalities. The gold standard for identifying engaging Hill–Sachs lesions and quantifying glenoid bone loss remains CT imaging. However, recent studies have shown that MRI is also capable of quantifying glenoid bone loss. Given that many surgeons will obtain an MRI to assess for soft-tissue injuries as part of their workup and preoperative planning, there may be a shift towards assessing glenohumeral bone loss via MRI in order to spare patients radiation exposure and reduce costs. Finally, some clinicians are beginning to incorporate ultrasound into their operative workup with promising results; however, this remains an emerging technique with limited high-quality evidence and heavy reliance on adept sonographers.
